# Insights into Bacterial Communities and Diversity of Mangrove Forest Soils along the Upper Gulf of Thailand in Response to Environmental Factors

**DOI:** 10.3390/biology11121787

**Published:** 2022-12-08

**Authors:** Pongrawee Nimnoi, Neelawan Pongsilp

**Affiliations:** 1 Department of Microbiology, Faculty of Liberal Arts and Science, Kasetsart University, Nakhon Pathom 73140, Thailand; 2 Department of Microbiology, Faculty of Science, Silpakorn University, Nakhon Pathom 73000, Thailand

**Keywords:** bacterial communities, mangrove forest soils, environmental factors

## Abstract

**Simple Summary:**

Mangrove forests are unique ecosystems located in tropical and subtropical tidal areas worldwide. In mangrove ecosystems, the bacterial community mediates nutrient transformation and therefore is essential for mangrove productivity and maintenance. The bacterial community structure in mangrove soil that is influenced by environmental factors thus merits comprehensive study. Illumina next-generation sequencing detected the unique biomarkers and predominant genera that established distinct niches in the mangrove soils along the Upper Gulf of Thailand. The bacterial diversity and community structure of the Mae Klong Estuary site were most dissimilar to those of the other sites, while the bacterial communities of Chaopraya Estuary, Laemphakbia Promontory, and Pranburi forest park sites were closer to each other. The Kungkrabaen Bay and Black Sand Beach sites had bacterial communities which were closest to each other. The mangrove soils were found variable with respect to pH and had low amounts of organic matter (OM). Soil OM was the major factor that modulated the bacterial community structure. The groups of ammonia-oxidizing, sulfate-reducing, and methanogenic bacteria were the significant biomarkers distributed in these mangrove soils.

**Abstract:**

The comprehensive data for the dynamic adaptation of bacterial community structure in response to environmental factors is important for the maintenance of the mangrove ecosystem. This aspect was investigated with soils and surface water from six mangrove forests in six provinces along the Upper Gulf of Thailand shoreline. Mangrove soils were variable with respect to pH (acidic to slightly alkaline) and had low amounts of organic matter (OM). Illumina next-generation sequencing attested that the number of observed species as well as the bacterial diversity and richness among all sites were not significantly different. The gamma-, alpha-*Proteobacteria*, *Desulfobacteria*, *Bacteroidia*, *Anaerolineae*, *Bathyarchaeia*, *Acidobacteriae*, *Nitrososphaeria*, *Clostridia*, and *Thermoplasmata* were more abundant bacterial classes present in all sites. Soil OM was the major factor that mostly modulated the bacterial community structure, while salinity influenced the number of observed species and bacterial richness. These results provide informative data on the bacterial community, in response to both environmental factors and heavy metal pollutants, that is prominent for sustainable development and management of mangrove forests.

## 1. Introduction

Mangrove forests are unique intertidal ecosystems, covering nearly 60–75% of the global tropical and subtropical coastline [1,2]. They are known as highly productive ecosystems which play important roles in the protection, stabilization, food supply, and habitation of aquatic organisms as well as the remediation of environmental pollution [2,3]. Mangrove forests are distinctive ecological niches [4] due to their quirky environmental conditions such as salinity level, OM content, and nutrient recycling rate, and consequently, become resource-rich habitats for microorganisms [5,6]. In addition, mangrove ecosystems are a treasure trove of natural product discovery, microbial diversity, and bioactivity survey [7,8]. Mangrove microbiota, which is composed of marine, freshwater, and terrestrial soil microorganisms, regulates many processes in the biogeochemical cycles such as nitrogen fixation, nitrification, denitrification, phosphate solubilization, carbon cycling, oxidation and reduction of sulfur and other elements as well as cellulose degradation [7,9,10]. Soil characteristics are one of the most important environmental factors that mainly affect mangrove productivity and structure [8]. Soil pH, EC, salinity, elements, and particle size are the major physicochemical properties of the mangrove soil that influence the chemical transformation of most nutrients and their availability to plants, resulting in incongruous ecological environments for diverse bacterial communities [3,11]. Therefore, it is merit to investigate the effect of soil and surface water characteristics of the mangrove forest soils on the bacterial community structure in various respects as well as the relationship between the bacterial diversity and richness and the elements in soil and surface water. This comprehensive study was conducted to revalorize the bacterial community structure and environmental factors associated with the mangrove forest soils, especially in the Upper Gulf of Thailand ecozone, where these aspects were unveiled. 

The semi-enclosed Gulf of Thailand is surrounded by four tropical countries: Thailand, Cambodia, Vietnam, and Malaysia [12,13]. The Upper Gulf has the shape of an inverted U letter, an area of approximately 10,000 square km, and a maximum depth of approximately 40 m. The Upper Gulf is adjacent to the estuaries of four large rivers: the Chaopraya River, the Thachin River, the Bangpakong River, and the Mae Klong River [14]. In Thailand, the mangrove forest area, estimated to cover approximately 229,618.56 ha, acts as a buffer between marine and terrestrial ecosystems [15,16]. Mangrove forests around the Upper Gulf of Thailand are muddy tidal flats on the coast and span along the eastern and southern coastlines. The common and dominant mangrove tree species on the coast of the Upper Gulf of Thailand include *Rhizophora apiculata*, *Rhizophora mucronata*, *Avicennia alba*, *Avicennia officinalis*, *Bruguiera cylindrica*, *Bruguiera parviflora*, *Ceriops decandra*, and *Ceriops tagal* [16]. Mangrove soils are formed by the accumulation of several components: (i) sediment that is derived from coastal and shore erosion; (ii) eroded soil from higher areas that flows downward in rivers and canals; (iii) sediment that is composed of colloidal materials and particles; and (iv) degraded OM. There are differences in sediment texture. River and canal sediment is fine and clayey, while coastal sediment is sandy [17,18]. The inappropriate land use of mangrove forests along the Upper Gulf of Thailand would cause coastal erosion, especially found in Samutprakan, Samutsakhon, Samutsongkhram, Phetchaburi, and Chachoengsao provinces [16,17,18]. The increases in urbanization, aquaculture, agriculture, industrialization, and pollution are major causes of mangrove forest degradation. Multifaceted knowledge of this important ecosystem is critical to forest management and rehabilitation, afforestation, and also resource conservation. Therefore, the insightful information regarding the association of bacteria, especially those that are largely involved in nutrient and biogeochemical cycling, with soil and surface water physicochemical properties merits investigation and notably for mangrove areas in Thailand, which have never been studied before. 

By using next-generation sequencing (NGS) technology, Hu et al. [3] found that the *Proteobacteria*, *Chloroflexi*, *Firmicutes*, and *Bathyarchaeota* were the dominant bacterial groups in the mangroves of south China and soil Cr, Ni, Cu, Zn, and Pb were the major factors affecting the bacterial diversity. Soil mineral compositions, especially Ca, Ti, Cu, and Zn, had strong correlations with the diversity and communities of bacteria associated with the roots of halophytes native to an Indonesian coastal sand dune [19]. *Proteobacteria*, *Firmicutes*, *Actinobacteria*, *Bacteroidetes*, and *Cyanobacteria* were the widespread, dominant phyla in Indian mangrove surface water. The prevalence of As, Ni, and Cu resistance genes was similar among the bacterial communities of surface water from all mangrove sites [20]. Based on its massive data generation, this study employed Illumina NGS of the V4 variable region of the 16S rRNA gene to investigate the mangrove soil bacterial communities at six sites in six provinces along the Upper Gulf of Thailand and to characterize the stratified bacterial communities and their compositions and diversity patterns in relation to the soil and surface water physicochemical factors. The results provide new insights into the relationship between the bacterial community and the soil and surface water physicochemical parameters associated with mangrove forests in Thailand. The derived multifaceted knowledge may be helpful for forest management and rehabilitation, afforestation, and also resource conservation in this precious ecosystem. 

## 2. Materials and Methods

### 2.1. Sample Collection 

**Figure 1:**
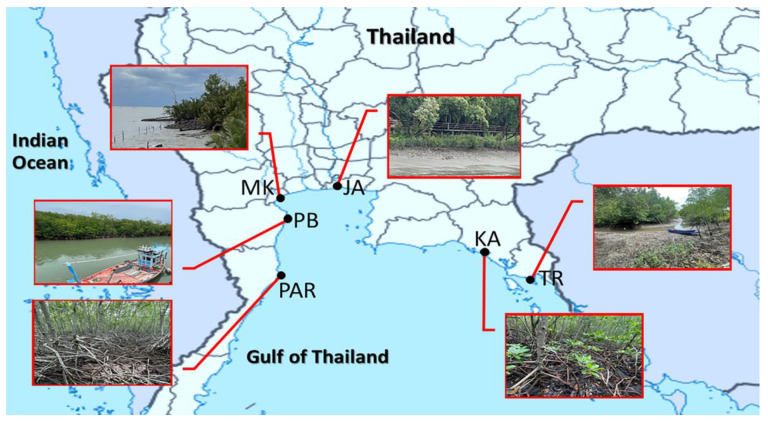
Map of sampling sites along the shores of the Upper Gulf of Thailand. The site codes are in accordance with those listed in Table 1. The photographs of sampling sites were taken by the authors.

Soils and surface water were sampled bilaterally on September 2021 from six mangrove forests in different provinces along the eastern, western, and southern coastlines of the Upper Gulf of Thailand. The sampling was carried out during high tide periods (10:00 a.m.–3:00 p.m.). The study sites were located at a distance of approximately 660 km (Table 1 and Figure 1). The study sites were localized at Black Sand Beach, which is a limonite-sand beach, Kungkrabaen Bay, which is a semi-enclosed bay, Chaopraya Estuary, Mae Klong Estuary, Laemphakbia Promontory, and Pranburi forest park, which is a beach forest. All mangrove forests in this study are mature forests. The common mangrove plant species in all sites were *Rhizophora apiculata* BI., *Rhizophora mucronata* Poir., and *Avicennia alba*. The common mangrove understorey species in all sites were *Ceriops decandra* (Griff.) Ding Hou, *Acrostichum aureum* L., and *Acanthus ebracteatus* Vahl. These mangrove forests have been affected by different categories of land use, including urbanization, aquaculture, agriculture, and recreation.

Soil samples were randomly collected at a 0 to 10 cm depth and a weight of 6 kg per site according to Li et al. [21] and mixed thoroughly. The mixed soil sample was divided into two portions. For the first portion, 20 g of soil was preserved with Sample-NAP Seq stabilization solution (Bioentist, Bangkok, Thailand), immediately stored in an ice box, and subjected to soil DNA extraction. For the second portion, soils were kept in sterile polyethylene bags, immediately stored in ice boxes, and subjected to soil physicochemical analyses. Once arrived at the laboratory, soil samples of both portions were immediately stored in a −80 °C fridge. For surface water collection, surface water in a volume of 1 L per site was collected, immediately stored in ice boxes, and used for the determination of physicochemical parameters. All sampling sites were public areas thus no specific authorization was required for sample collection. 

### 2.2. Determination of Soil and Water Physicochemical Parameters

For soil physicochemical analyses, 2 kg of soil from each sampling site were air-dried and sieved through a sterile 10-mesh sieve with a 2-mm aperture size. Soil EC and pH were measured as described by Jackson [22]. Soil particle size distribution was analyzed as described by Bouyoucos [23]. OM content was determined following the method of Walkley and Black [24]. Amounts of P, Fe, Ca, Cu, Mn, and Zn were determined by the Bray II method [25]. Amounts of K and Mg were analyzed by the Flame photometric method [22]. The quantity of N was analyzed as described by Li et al. [21]. For surface water parameter analyses, pH and NaCl concentration were measured by a benchtop pH meter (Metrohm 827 pH Lab, Herisau, Switzerland) and a refractometer (Atago N-1E, Tokyo, Japan) respectively, following the standard procedures of American Public Health Association (APHA) [26]. One hundred milliliters of each surface water sample was filtered using Whatman filter paper no. 1 and then 10 mL of 3% nitric acid (HNO_3_) was added into the filtrate [27]. Concentrations of 13 heavy metals in surface water (Cd, Cr, Ag, Se, Cu, Al, Ba, Hg, Mn, As, Zn, Pb, and Fe) were determined using an inductively coupled plasma-mass spectrometry (ICP-MS) (Agilent ICP-MS 7900, Santa Clara, CA, USA) as described by Abdul et al. [28]. 

### 2.3. Soil DNA Extraction and Illumina NGS

Soil DNA of three replicates per sample was extracted by a NucleoSpin soil kit (Macherey-Nagel, Duren, Germany) following the manufacturer’s instructions. Then, the quantity and purity of the extracted DNA were determined with a DS-11FX+ Spectro/Fluorometer (DeNovix, Wilmington, DE, USA). A pair of primers, 515F and 806R, latched onto the barcodes [29,30] were used for PCR amplification of the V4 variable region of the 16S rRNA gene by using Phusion High-Fidelity PCR Master Mix (New England Biolabs, Ipswich, MA, USA). The PCR products were purified by a Qiagen gel extraction kit (Qiagen, Germantown, MD, USA). The libraries of amplified DNA were constructed with a TruSeq DNA PCR-free sample preparation kit (Illumina, San Diego, CA, USA) and sequenced with a HiSeq2500 PE250 sequencing platform (Illumina, San Diego, CA, USA) as described in the manufacturer’s instructions. Negative controls (reactions with sterile water) were carried out in parallel through amplification and sequencing.

### 2.4. Data Processing and Bioinformatics Analyses

Sequence reads were merged by the FLASH software version 1.2.7 (https://ccb.jhu.edu/software/FLASH/, accessed on 20 October 2022) [31]. The raw tags were then qualified by using the QIIME software version 1.7.0 (http://qiime.org/, accessed on 20 October 2022) [32] for the selection of the high-quality clean tags. The chimera sequences were detected and removed from the screened tags by using the UCHIME algorithm by comparing them with reference sequences retrieved from a database, resulting in the eventual effective tags [33]. For operational taxonomic unit (OTU) clustering and species annotation, all effective tags were bioinformatically processed with the Uparse software version 7.0.1001 (https://drive5.com/uparse/, accessed on 20 October 2022) [34]. Sequences sharing ≥97% similarity were assigned to the same OTUs. The Mothur software version 1.36.1 [35] was used for species annotation at each taxonomic level by sequence alignment of each representative sequence with reference sequences in the SSU rRNA database of SILVA [36]. The MUSCLE program version 3.8.31 (https://www.drive5.com/muscle/, accessed on 20 October 2022) [37] was used to depict the phylogenetic relationship of all OTUs derived from representative sequences. All resulting sequences derived in this study are assessable in the Sequence Read Archive of the National Center for Biotechnology Information (NCBI) under the BioProject accession number: PRJNA856803.

### 2.5. Statistical Analyses

The QIIME software version 1.7.0 (http://qiime.org/, accessed on 20 October 2022) [32] was used to compute the parameters relating to alpha diversity, including community diversity (Shannon–Weaver and Simpson’s indices), community richness (Chao1 and ACE estimators), and index of sequencing depth (Good’s coverage) as well as beta diversity to quantify sample variations in species complexity. The analyzed data was displayed using the R software version 2.15.3 (https://www.r-project.org/, accessed on 20 October 2022) [38]. Principal coordinate analysis (PCoA) was performed to visualize complex, encompassed data, which was then displayed by the WGCNA, stat, and ggplot2 packages [39] in the R software version 2.15.3 (https://www.r-project.org/, accessed on 20 October 2022) [38]. The unweighted-pair group method with arithmetic mean (UPGMA) clustering, which was conducted as a type of hierarchical clustering method to interpret the distance matrix using average linkage, was operated using the QIIME software version 1.7.0 (http://qiime.org/, accessed on 20 October 2022) [32]. Linear discriminant analysis (LDA) effect size (LEfSe) analysis that is accompanied in the LEfSe software version 1.1.0 (https://huttenhower.sph.harvard.edu/lefse/, accessed on 20 October 2022) [40] was applied to identify differentially abundant groups among samples. The nonparametric method, analysis of molecular variance (AMOVA), was calculated by the Mothur software version 1.36.1 (https://mothur.org/, accessed on 20 October 2022) [35]. Analysis of similarity (ANOSIM) was computed by the R software version 2.15.3 (https://www.r-project.org/, accessed on 20 October 2022) [38] to assess significant inter- and inner-group differences among bacterial community structures. 

The canonical correlation analysis (CCA) was conducted by using the PAST software version 4.03 (https://palaeo-electronica.org/2001_1/past/issue1_01.htm, accessed on 20 October 2022) [41] to determine associations among biotic and abiotic factors. Soil and surface water physicochemical parameters and alpha diversity indices were included in analysis of variance (ANOVA) using Tukey’s test. The association of soil and water physicochemical parameters with bacterial communities was assessed based on Spearman’s correlation. Statistical significance was indicated by *p*-value (very significant if *p*-value was < 0.01 and significant if *p*-value was < 0.05). The parameter whose *p*-value was < 0.01 and < 0.05 was considered the major and minor factor, respectively. Between-group analysis, ANOVA, and Spearman’s correlation were performed with the SPSS software version 19.0 (IBM Corp., Chicago, IL, USA). All data analyses were performed with three replicate samples.

## 3. Results

### 3.1. Soil and Surface Water Physicochemical Parameters

Soils and surface water were bilaterally collected from six mangrove forests in different provinces along the Upper Gulf of Thailand. The geographical locations of the six sampling sites are shown in Table 1 and Figure 1. The physicochemical parameters of mangrove soil samples are shown in Table 2. Among all sampling sites, pH and EC average values ranged from 4.98 to 7.40 and 12.33 to 44.51, respectively. Site KA (at Kungkrabaen Bay) had the significantly highest values of most (five) physicochemical parameters, including OM, total N, total P, total K, and total Mg, whereas it contained the significantly lowest amount of total Mn. Site JA (at Chaopraya Estuary) exhibited the significantly highest amounts of total Mn, total Cu, and total Zn, while it had the significantly lowest EC value. Site PAR (at Pranburi forest park) had the significantly highest measures of EC and total Fe, though it had the significantly lowest values of pH, total K, and total Ca. Sites PB (at Laemphakbia Promontory) and MK (at Mae Klong Estuary) had the significantly highest measures of pH and total Ca, respectively. Sites PB, MK, and PAR shared the lowest rank of total Mg which was statistically equal to each other. Besides total Mg, site PB had the significantly lowest contents of five other parameters, including OM, total N, total Fe, total Cu, and total Zn. Lastly, site TR (at Black Sand Beach) had the significantly lowest amount of total P. Moreover, four different soil physical properties were sorted based on the proportions of sand, silt, and clay. Sites TR, JA, and PB were in the category of silt loam soil. Sites KA, MK, and PAR were fitted into different soil types: silty clay loam soil, loam soil, and sandy loam soil, respectively.

The physicochemical characteristics of surface water from each sampling site were investigated. As shown in Table 3, among all sampling sites, pH values and NaCl concentrations ranged from 7.53 to 8.01 and 0% to 3%, respectively. Sites MK and KA had the significantly highest and significantly lowest pH values, respectively. NaCl could not be measured in surface water from sites TR, JA, and MK, whereas site PB contained the significantly highest level of NaCl at 3%, followed by sites PAR and KA, respectively. Among 13 heavy metals which were quantified, the amounts of total Cd, total Cr, total Se, total Hg, and total Fe could not be detected or were below the lowest limits of quantitation in all sites. Site TR had amounts of total Cu which were detected only in this site, total Al and total Zn which were significantly lowest as well as total Mn. Site KA contained the amounts of total Ag and total Al which were significantly highest, total Ba which was significantly lowest, and total Mn. Site MK contained the amounts of total Ba, total Mn which was significantly lowest, total As which was detected only in this site, and total Zn which was significantly highest. Site PB had total Ag which was significantly lowest, total Al, total Ba, and total Pb. Site JA contained the amounts of total Ba, total Mn which was significantly highest, and total Pb which was significantly lowest. Site PAR contained amounts of total Al which were significantly lowest as well as total Ba and total Pb which were significantly highest. 

### 3.2. Sequence Analyses and Bacterial Diversity Indices

**Figure 2:**
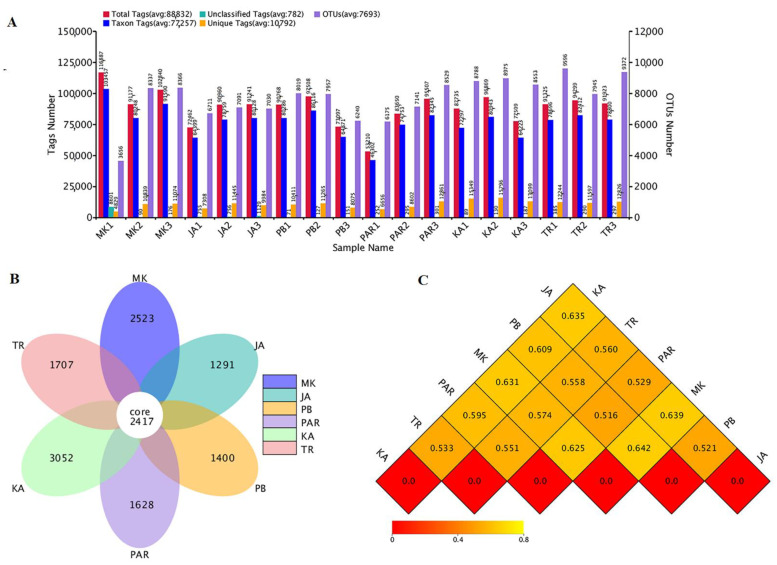
Statistical analyses on bacterial community structure differentiation. Tag and OTU numbers of each sampling site (**A**); OTUs flower diagram (**B**); heat map showing dissimilarity coefficients obtained from pairwise comparisons between sampling sites (**C**). The site codes are in accordance with those listed in Table 1.

A total of 2,181,870 raw reads obtained from 18 DNA samples (three replicates/site) were subjected to tag merge and sequence quality control, resulting in a total of 2,118,867 qualified tags (97.11% of raw reads). Removal of potential chimera tags from qualified tags by the UCHIME algorithm yielded a total of 1,390,625 taxon tags. The tags sharing ≥97% sequence similarity were grouped into the same OTUs. A total of 28,052 OTUs were obtained from all samples, with a mean Good’s coverage of 95.23 ± 1.50%. The numbers of total tags, taxon tags, unclassified tags, unique tags, and OTUs from each replicate are shown in Figure 2A. As presented in a Flower display (Figure 2B), 2417 OTUs were common in all sampling sites. Site KA (at Kungkrabaen Bay), that exhibited the significantly highest values of five soil physicochemical parameters, had the highest unique OTUs (3052 OTUs), followed by sites MK (at Mae Klong Estuary), TR (at Black Sand Beach), PAR (at Pranburi forest park), PB (at Laemphakbia Promontory), and JA (at Chaopraya Estuary), respectively.

In addition, the alpha diversity parameters including diversity indices (Shannon–Weaver and Simpson) and richness indices (Chao1 and ACE) as well as the number of observed species in each sampling site were evaluated (Table 4). Among all sampling sites, the numbers of observed species varied from 5814.00 ± 2415.35 to 7563.66 ± 657.49. Raw sequence and taxonomically classified sequence data of triplicate samples are shown in Supplementary Table S1. The rank abundance curve of observed species in mangrove forest soils (Supplementary Figure S1) ensured normal data and thus confirmed the correctness and precision of species annotation. Site TR had the highest number of observed species, followed by sites KA, PAR, PB, JA, and MK, respectively, although the numbers of observed species in all sites were not significantly different from each other. As higher values of Shannon–Weaver and Simpson indices signify higher bacterial diversity, sites KA and MK had the highest and lowest bacterial diversity, respectively, according to the Shannon–Weaver index. There was no significant difference in bacterial diversity among all sites. Simpson index similarly connoted that site MK had the lowest bacterial diversity which was not significantly different from that of other sites. Site TR had the highest bacterial richness, based on richness indices (Chao1 and ACE), whereas site JA exhibited the lowest bacterial richness which was not significantly different from that of other sites. Moreover, a heat map (Figure 2C) which displays the dissimilarity coefficients between pairwise samples revealed that the bacterial compositions of sites PB and PAR were most similar to each other. On the contrary, the bacterial compositions of sites MK and PB were most dissimilar to each other. Site TR which contained the highest number of observed species and bacterial richness was most similar to site KA which had the highest bacterial diversity, though most dissimilar to site MK which had the lowest number of observed species, and the lowest bacterial diversity and JA which had the lowest bacterial richness. 

The correlations between soil physicochemical parameters and bacterial diversity and richness were characterized. The results show that site TR, in which soil contained the significantly lowest amount of total P, had the highest number of observed species and the highest bacterial richness. Site KA, in which soil contained the significantly highest values of OM, total N, total P, total K, and total Mg, had the highest bacterial diversity. Whereas, site MK, in which soil contained the significantly highest amount of total Ca, had the lowest number of observed species and the lowest bacterial diversity. Site JA containing the significantly highest amounts of total Mn, total Cu, and total Zn had the lowest bacterial richness.

### 3.3. Illumina NGS and Bacterial Community Structure

**Figure 3:**
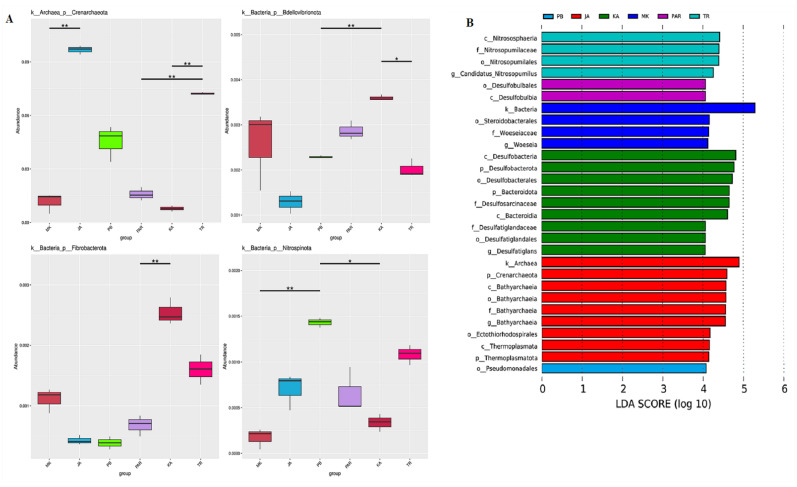
Between-group variation analyses. Bar chart showing the abundance of bacterial phyla in each sampling site (**A**). A double asterisk represents a highly significant variation (*p* < 0.01), and a single asterisk represents a significant variation (*p* < 0.05); histogram of LDA scores for evaluation of biomarkers whose numbers were statistically different among sampling sites (**B**). The site codes are in accordance with those listed in Table 1.

Among the top ten most abundant phyla present in all samples, the *Proteobacteria* was the most abundant, ranging between 22.34 and 37.11%, followed by the *Desulfobacterota* (6.71–17.62%), *Bacteroidota* (3.47–12.36%), *Chloroflexi* (7.07–12.02%), *Crenarchaeota* (0.78–9.67%), *Acidobacteriota* (3.30–8.88%), *Firmicutes* (0.34–6.21%), *Myxococcota* (1.98–3.90%), *Gemmatimonadota* (2.50–3.83%), and *Halobacterota* (0.00–3.73%). A tiny proportion of sequences from the archaeal phylum *Crenarchaeota* was derived because it also harbors the V4 variable region of the 16S rRNA gene. The statistically distinguishable variations in phylum among samples, according to the observed abundance, were elucidated (Figure 3A).

**Figure 4:**
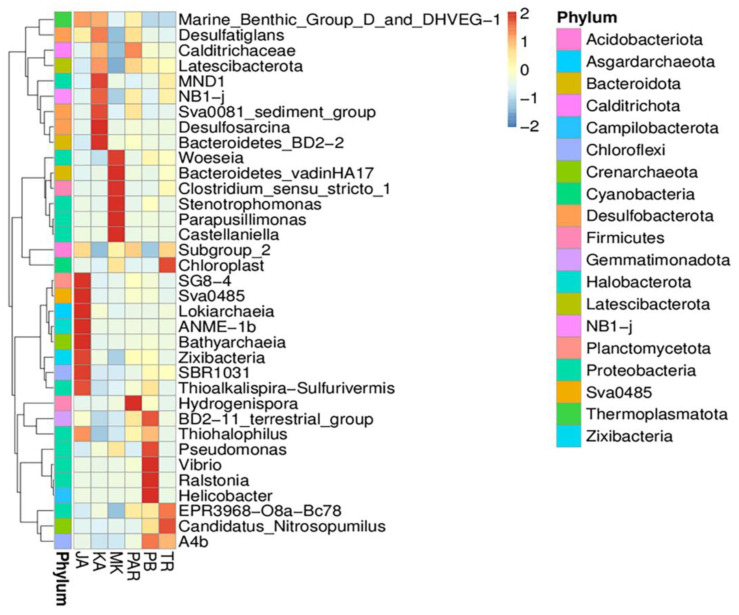
Heat map analysis of genus distribution in each sampling site. The site codes are in accordance with those listed in Table 1.

The results show that the phylum *Crenarchaeota* was very significantly variable (*p* < 0.01) between sites JA and MK and among sites TR, KA, and PAR. The phylum *Bdellovibrionota* was very significantly variable among sites KA, PB, and PAR, and significantly variable (*p* < 0.05) between sites TR and KA. The phylum *Fibrobacterota* was very significantly variable between sites KA and PAR. The phylum *Nitrospinota* represented highly significant variations when compared among sites JA, MK, and PB, and significant variations among sites KA, PB, and PAR. To obtain a finer evaluation of the unique bacterial community characteristic in each sampling site, a biomarker analysis was conducted. The LDA scores derived from the output of a biomarker analysis represented significant differences in the abundance of bacterial classes among sites (Figure 3B). The classes *Nitrososphaeria* and *Desulfobulbia* were significantly more abundant in sites TR and PAR, respectively. The classes *Desulfobacteria* and *Bacteroidia* were significantly more abundant in site KA. The classes *Bathyarchaeia* and *Thermoplasmata* were significantly more abundant in site JA. To further analyze the distribution of each genus among all sampling sites, a heat map analysis was conducted. The different colors in a heat map chart (Figure 4) signify the levels of abundance and the distribution of genera in the bacterial communities. The colors which shade from deep blue into dark brown denote low to high levels of relative abundance. The most abundant genera in each site are marked as dark-brown squares in a heat map chart. The SG8-4, Sva0485, *Lokiarchaeia*, ANME-1b, *Bathyarchaeia*, *Zixibacteria*, SBR1031, *Thioalkalispira*, and *Sulfurivermis* were the predominant genera in site JA. The *Desulfatiglans*, MND1, NB1-j, Sva0081 sediment group, *Desulfosarcina*, and *Bacteroidetes* were more abundant than other genera in site KA. The *Woeseia*, *Bacteroidetes*, *Clostridium*, *Stenotrophomonas*, *Parapusillimonas*, and *Castellaniella* were more common in site MK. Site PAR had *Calditrichaceae* and *Hydrogenispora* as the highly abundant genera. The BD2-11 terrestrial group, *Pseudomonas*, *Vibrio*, *Ralstonia*, *Helicobacter*, and A4b, were more abundant than other genera in site PB. The chloroplast, EPR3968-O8a-Bc78, *Candidatus*, and *Nitrosopumilus* were more common in site TR. 

**Figure 5:**
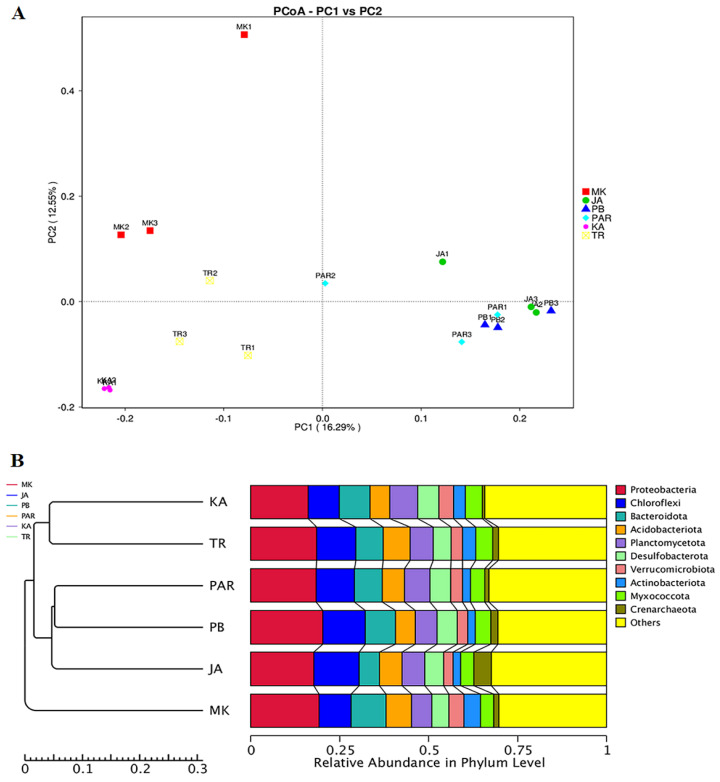
Clustering of bacterial communities. Principal coordinate analysis (PCoA) of bacterial composition similarity according to sampling site (**A**); UPGMA dendrogram of relative abundance at phylum level form each sampling site (**B**). The site codes are in accordance with those listed in Table 1.

The AMOVA method revealed significant variations in bacterial community structure among all sampling sites (Fs = 6.859; *p* < 0.001). This result was additionally confirmed by the ANOSIM which indicated that the variations of inter-group bacterial community structure were larger than those of the inner-group (R = 1). The nonparametric multivariate variance test according to distance matrix (ADONIS), which was used for estimating the significance of grouping among samples, indicated that the bacterial community of site PAR was closer to that of sites PB (R_2_ = 0.501; *p* = 0.001) and JA (R_2_ = 0.524; *p* = 0.001), respectively. The bacterial community of site KA was closer to that of site TR (R_2_ = 0.664; *p* = 0.001) than that of site MK (R_2_ = 0.406; *p* = 0.001). The ordination of sampling sites by PCoA shown in Figure 5A illustrated the alienated bacterial community of site MK, while the bacterial communities of sites JA, PB, and PAR were closer to each other. Sites KA and TR had bacterial communities which were closest to each other. The clustering analysis for determining the similarity among different samples was performed based on the UPGMA hierarchical clustering method (Figure 5B). The results also demonstrate the closer relations among bacterial communities in sites JA, PB, and PAR. Sites KA and TR had bacterial communities which were closest to each other, whereas that of site MK was plotted apart from other sites. These results reveal the unique biomarkers and predominant genera that established a niche in each sampling site as well as indicate the differences in bacterial community structure among sampling sites, in which sites JA, PB, and PAR were more similar to each other than were sites KA and TR, and MK. 

### 3.4. Effect of Environmental Factors on the Distribution of Bacterial Community Structure

The effect of soil and surface water physicochemical parameters on the distribution of bacterial communities was investigated. The results (Table 5) exhibit that the gamma-*Proteobacteria* was significantly positively associated with soil pH, and significantly negatively associated with soil OM, total soil N, total soil Mg, total soil Fe, total soil Cu, total soil Zn, and total water Mn. The *Desulfobacteria* was significantly positively associated with soil EC, soil OM, total soil P, total soil Fe, and total water Al, and significantly negatively associated with soil pH, total soil Ca, total soil Mn, water pH, total water As, and total water Zn. Members of *Bacteroidia* and *Clostridia* were significantly positively associated with soil EC and total water As, respectively. *Anaerolineae* was significantly positively associated with soil pH and total soil Mn, and significantly negatively associated with soil OM and total soil Fe. Members of alpha-*Proteobacteria*, *Acidobacteriae*, and *Nitrososphaeria* were significantly negatively associated with total soil P, total soil P, and total soil Zn, respectively. *Bathyarchaeia* was significantly positively associated with total soil Mn and total water Pb, and significantly negatively associated with total soil K. *Thermoplasmata* was significantly positively associated with total soil P, total soil Mg, total soil Fe, total soil Cu, and total soil Zn, and significantly negatively associated with total water Zn. The bacterial community was significantly positively associated with soil OM and total soil N, and significantly negatively associated with soil pH and total soil Mn. The bacterial richness and number of observed species were significantly negatively associated with total water Pb. 

Moreover, percentages of OTUs of the top ten most abundant bacterial classes present in each sampling site were compared (Table 6). Site TR presented the highest number of *Nitrososphaeria* which was significantly different from that in other sites. Site KA had the highest numbers of the *Desulfobacteria* and *Bacteroidia*, and the lowest numbers of the gamma-*Proteobacteria*, *Anaerolineae*, alpha-*Proteobacteria*, *Bathyarchaeia*, *Acidobacteriae*, *Nitrososphaeria*, and *Clostridia*. Site JA had significantly highest numbers of *Bathyarchaeia* and *Thermoplasmata*, whereas it had the lowest number of *Bacteroidia* which was not significantly different from that in sites TR, PB, and PAR. Site MK had the highest and lowest numbers of *Clostridia* and *Desulfobacteria*, respectively. Site PB had the highest numbers of gamma-*Proteobacteria* and *Anaerolineae*, and the lowest number of *Thermoplasmata*. Site PAR had the highest number of *Acidobacteriae*, though it was not significantly different from that in other sites.

To definitively reveal the relationship between environmental factors and bacterial diversity and richness, the CCA was analyzed (Supplementary Figure S2A). The results reveal that soil parameters including total K, total N, and OM were more closely related in the same direction to the bacterial diversity. However, NaCl was the only factor that was closely related in the same direction to the number of observed species and bacterial richness, though there was no statistically significant correlation between either diversity or richness and NaCl (*p* > 0.05). Despite the fact that NaCl had no statistically significant effect on bacterial diversity and richness, it might affect the distribution of dominant bacterial taxa in soils. In order to comprehend the effect of NaCl on the distribution of dominant bacteria taxa, ternary plots were drawn to astutely distinguish the relative abundance of the top ten most abundant bacterial classes between the presence and absence of NaCl (Supplementary Figure S2B,C). The results show that in sites KA, PB, and PAR where NaCl was present in surface water, gamma-*Proteobacteria* was the dominant class that was mostly distributed in the soil environment and was most closely associated with the *Anaerolineae*. In comparison with the presence of NaCl, the ternary plot of sites TR, JA, and MK, where NaCl was absent, shows that gamma-*Proteobacteria* was also the dominant class that mostly inhabited the soil environment but most closely associated with *Acidobacteriae* and alpha-*Proteobacteria* instead of *Anaerolineae*, as observed in sites containing NaCl. Comparison of the circle sizes illustrated in both ternary plots represents that NaCl also vividly altered the relative abundance of specific bacterial classes. The presence of NaCl increased the relative abundance of *Desulfobacteria* and *Bacteroidia*, though decreased that of *Bathyarchaeia*, *Acidobacteriae*, and *Nitrososphaeria*. In this study, when evaluating the factor affecting the bacterial community structure, soil OM and soil total N was the major factor (*p* < 0.01) and the minor factor (*p* < 0.05), respectively.

## 4. Discussion

Mangrove forests are important ecosystems distributed in tropical and subtropical tidal areas worldwide [3]. Within the mangrove ecosystems, microorganisms are the main players that regulate the mangrove ecosystems by transformation and recycling of major nutrients, thus they are essential to the productivity, conservation, and rehabilitation of mangroves [42,43]. Bacteria and fungi constitute the lion’s share (91%) of the total biomass, while algae and protozoa account for 7% and 2%, respectively, in mangrove ecosystems [42]. A variety of bacteria are responsible for most biogeochemical cycles and a process of energy flow in tropical mangrove sediments [8].

Mangroves are ecosystems that have variable physicochemical conditions, including salinity, pH, soil grain size, and contaminant [3]. In the present study, soil pH values ranged from 4.98 to 7.40, indicating that the mangrove soils along the Upper Gulf of Thailand varied from acidic to slightly alkaline. The variations in the pH value of the mangrove soils within the same areas were previously reported. The pH values of mangrove soils in a Bhitarkanika mangrove, India, and Mai PO Nature Reserve, China, varied from 6.02 to 7.89 [44] and from 5.82 to 8.17 [45], respectively. The mangrove soil of Qua Iboe Estuary, Nigeria, which experienced oil spillage, was acidic (a mean pH of 6.36 during the wet season) [46]. The mangrove forest soil in Balandra Beach, Mexico, which is an arid region, was slightly alkaline (pH of 7.80) [47]. The rhizosphere soil of a mangrove species (*Avicennia marina*) on the Thuwal Coast of the Red Sea, Saudi Arabia, was alkaline (a mean pH of 8.14 ± 0.6) [48]. The pH values of mangrove soils varied depending on several factors such as geographical region, season, aridity, mangrove species, and pollution. Soil pH influences the bacterial community structure whereby it affects the chemical transformation of most nutrients and their availability to plants. Since then, unique ecological environments for diverse bacterial communities have been established [11]. A decrease in pH resulted in a higher number of acidophiles and a lower number of mesophiles or alkalophiles [3,45].

In this study, the variable concentrations of soil OM among sampling sites were observed. The soil OM concentrations ranged from 0.19 to 1.85 g/kg, and they were significantly positively associated with soil elements including total N, total K, total Mg, total Fe, and total Cu. The amounts of OM in the sites of this study were much lower when compared to those in other reports. For example, the amounts of OM in the mangrove soils in Sanya Mangrove Nature Reserve, China, were between 23.8 and 102.8 g/kg [49]. The mangrove soil in Cardoso Island, Brazil, had OM concentrations ranging between 27 and 36 g/kg [4]. The Barra Grande mangrove soil in Northeastern Brazil had OM at concentrations ranging between 24 and 84 g/kg [50]. The different amounts of OM probably resulted from the differences in biological composition and location, such as plant species, runoff water, and anthropogenic contaminants, that are attributes for the replenishment of unique environments [8,44]. The increases in the amount of OM and nutrients might be attributed to the enhanced decomposition of leaf and root litter [45].

The amounts of heavy metals in mangrove soils and surface water were bilaterally determined in this work. We found that the average concentrations of heavy metals in soils ranking from highest to lowest were Fe (223.78 mg/kg) > Mn (97.21 mg/kg) > Zn (6.60 mg/kg) > Cu (5.48 mg/kg). This ranking corresponds to the report of Lertprasert [51] who found that the concentrations of metals in the sediment of the Phi Lok Canal system of Samutsongkhram province, Thailand, were ranked from highest to lowest as follows: Fe > Zn > Cu. The heavy metals in mangrove sediments were mainly from industrial waste, urban runoff, shipbuilding, chemical dumping, and leaks from mineral operations [52,53]. Sediment texture, OM, and cation exchange capacity were the factors responsible for heavy metal accumulation. The concentrations of heavy metals were found to be higher inland and gradually reduced along the closer distance to the sea [16]. Moreover, heavy metals, such as Cr, Cd, Hg, and Pb, which are dissolved in aquatic environments, are potentially hazardous and can be absorbed easily by living organisms, consequently, entering the food chain. Their accumulation in the human body raises the concern for baneful impacts of heavy metals [54]. According to the permissible limits of heavy metals in wastewater (effluent) recommended by the U.S. Environmental Protection Agency (EPA) [55] and the World Health Organization (WHO) [56], the levels of 12 heavy metals (Cd, Cr, Ag, Se, Cu, Al, Ba, Hg, As, Zn, Pb, and Fe) in surface water from all sites passed both standard criteria except the level of Mn in site JA (at Chaopraya Estuary), that was higher than the safe limit of WHO (0.2 mg/L). Manganese poisoning in humans causes neurodegenerative diseases such as Alzheimer’s disease, Huntington’s disease, and amyotrophic lateral sclerosis [57]. Therefore, a suitable management program and monitoring system should be applicable to assess heavy metal levels in these natural sites.

In this study, we also found the relations of soil parameters, including OM, total N, total P, total K, total Mg, total Fe, and total Cu, to the soil bacterial diversity in each sampling site (Supplementary Figure S2A). This finding is in agreement with those of Mishra et al. [44] and Rojas et al. [58], in which the relatively more amounts of some nutrients, such as N, P, and K, would alter the microbial compositions by promoting the soil populations of heterotrophic, phosphate-solubilizing, and sulfur-oxidizing bacteria.

Interestingly, the effect of soil and surface water physicochemical parameters on the distribution of bacterial communities was investigated. Soil physicochemical properties influenced the bacterial communities to varying degrees. Soil OM was the major factor and soil total N was the minor factor that modulate the bacterial community structure. The impact of environmental factors on coastal and marine bacterial communities was reported in previous studies. Soil mineral compositions, including Ca, Ti, Cu, and Zn, were validated as the main factors that shape the bacterial communities associated with the roots of halophytes native to an Indonesian coastal sand dune [19]. The bacterial communities of seawater along the Upper Gulf of Thailand coastline were mainly determined by salinity, total N, and total P [13]. The structures of sedimentary bacterial communities in the Chinese mangroves were dependent on several environmental factors such as pH, salinity, OM, and metal pollution [3]. The impact of heavy metals on bacterial diversity and community was reported. Hu et al. [3] found that heavy metals such as Pb had a significant negative impact on the bacterial communities and diversity of mangrove Reserves in China. This result is consistent with our result demonstrating that surface water Pb was significantly negatively associated with the bacterial richness and number of observed species. Among the ten heavy metals examined, As and V mostly contributed to shaping the bacterial communities in sediments from lakes with intensive fish aquaculture in Peru [59]. Bioavailable heavy metals could affect the composition and activity of bacterial communities whereby high amounts of heavy metals inhibited the growth of some bacterial taxa [60,61]. Soil minerals could affect bacterial growth in a taxon-specific manner. The association of minerals with bacteria appeared to influence biogeochemical cycling by altering nutrient availability in a mangrove environment [19].

The metagenomics data of mangrove soils in this study revealed that the numbers of observed species and bacterial diversity and richness in all sampling sites were not significantly different from each other, mainly due to the similarity in dominant tree species and mixed land use. All sites were utilized in different categories of land use, including urbanization, aquaculture, agriculture, and recreation. The phylum *Proteobacteria* was the most abundant, followed by *Desulfobacterota*, *Bacteroidota*, *Chloroflexi*, *Crenarchaeota*, *Acidobacteriota*, *Firmicutes*, *Myxococcota*, *Gemmatimonadota*, and *Halobacterota*. This result is similar to those reported in the previous studies. For example, Liu et al. [7] reported that *Proteobacteria* was the most abundant in the soil samples of the Bamenwan mangrove forest, China, followed by *Actinobacteria*, *Chloroflexi*, *Acidobacteria*, *Firmicutes*, *Bacteroidetes*, *Gemmatimonadetes*, *Cyanobacteria*, *Nitrospirae*, and *Chlorobi*. Ghosh et al. [20] found that *Proteobacteria* was the most abundant in the Indian mangrove ecosystem, followed by *Firmicutes*, *Bacteroidetes*, and *Actinobacteria. Proteobacteria*, *Firmicutes*, *Actinobacteria*, *Bacteroidetes*, and *Chloroflexi* were the most abundant phyla, in decreasing order, in the mangrove soil samples in Sao Paulo State, Brazil [62]. *Proteobacteria* were the dominant phylum, followed by *Chloroflexi*, across all four sampling sites in the mangrove Reserves of South China [3]. These results support that *Proteobacteria* was the most predominant bacterial phylum and became cosmopolitan bacteria in diverse mangrove environments.

Even though the most dominant phyla in all sites were obviously similar, the differences in relative abundance between bacterial groups of sampling sites could be detected. These differences possibly resulted from different biogeographical and anthropogenic factors such as urbanization, mangrove plant composition, and proximity to aquaculture farms [7]. General physiological conditions, such as oxygen level and a variety of biomacromolecules that were distributed in different forms and concentrations throughout the individual aquatic and terrestrial environments, significantly supported the propagation of different dominant bacterial groups [45]. The members of *Proteobacteria* are metabolically diverse and can sustain a wide range of ecosystems because they harbor intricate groups of genes responsible for stress resistance, then enhancing their adaptive capabilities and survival that are attributes of dominant groups [20,63]. *Proteobacteria*, *Bacteroidetes*, and *Chloroflexi*, which are usually the dominant taxa in mangrove and terrestrial environments, are consistently important contributors to biogeochemical processes, such as nitrogen fixation, ammonification, nitrification, denitrification, carbon fixation, carbon degradation, phosphate solubilization, sulfite reduction as well as nitrogen, phosphorus, and iron acquisition [64,65,66,67]. The high amount of *Firmicutes* contributed to the activity of sulfur-oxidizing bacteria which detoxified sulfide to reduce pollution in the mangrove ecosystem [68].

Moreover, after conducting a biomarker analysis, we found significant differences in the dominant bacterial class distributed in sampling sites. The classes *Nitrososphaeria* and *Desulfobulbia* were significantly more abundant in sites TR (at Black Sand Beach) and PAR (at Pranburi forest park), respectively. The classes *Desulfobacteria* and *Bacteroidia* were significantly more abundant in site KA (at Kungkrabaen Bay). The classes *Bathyarchaeia* and *Thermoplasmata* were significantly more abundant in site JA (at Chaopraya Estuary). The orders *Steroidobacterales* (especially the genus *Woeseia*) and *Pseudomonadales* were more abundant in sites MK (at Mae Klong Estuary) and PB (at Laemphakbia Promontory), respectively. These dominant taxa drive processes in the biogeochemical cycles. *Nitrososphaera* is a genus of ammonia-oxidizing archaeans that belongs to the class *Nitrososphaeria* and was found widespread and in large amounts in almost all terrestrial and marine environments. *Nitrososphaera* plays a major role in nitrification, resulting from the high expression of *amoA* or *amoA*-like genes that contribute to the potential of bulk nitrification [69]. This assumption was supported by the heat map result in which ammonia-oxidizing bacterial genera, *Candidatus* and *Nitrosopumilus*, were more common in site TR. The class *Desulfobulbia*, which was dominant in site PAR, has the activity of reducing sulfate to sulfide for obtaining energy under anaerobic conditions. Members of this class transported electrons from the hydrogen sulfide-rich sediment to the oxygen-rich sediment that was in contact with water in the top layer of marine sediment [70,71,72]. Members of the class *Desulfobacteria*, which was dominant in site KA, is a group of sulfate-reducing bacteria that use alternative electron acceptors in anaerobic respiration to form hydrogen sulfide [2]. Hydrogen sulfide emitted into the atmosphere is assimilated by anaerobic photosynthetic bacteria and sulfur-oxidizing bacteria [73]. The *Bacteroidia*, another dominant class in site KA, was reported for its attributes: (i) the capability to drive protein metabolism via proteolytic activity; (ii) the production of succinic acid and acetic acid; and (iii) the wide distribution in various environmental samples including soil, sediment, and seawater [74,75]. The dominant class in site JA, *Bathyarchaeia*, was reported for their occurrence in the methane-rich sediments across tropical coastal ecosystems in Brazil and their role in methanogenesis and carbon dioxide reduction linked to the carbon cycle [76,77]. Carbon dioxide fixation, sulfur reduction as well as formaldehyde and acetate assimilation were the predicted capability of the *Thermoplasmata*, another dominant class in site JA [78]. The order *Steroidobacterales*, the major population in site MK, was found to be common in beach ecosystems across the United States [79], Fe-Mn encrusted coral from the southwestern Atlantic Ocean [80], and Fe-Mn crust biofilm from the south Atlantic Ocean [81]. The genus *Woeseia* is the main inhabitant of mangroves in southern China [82], sediments from the Mariana Trench, the western Pacific Ocean [83], and marine oxygen minimum zones (OMZs) in the Namibian Shelf [84]. Its involvement in hydrocarbon degradation, nitrate assimilation, and sulfur oxidation was proposed [82,83,84]. Some members of the *Pseudomonadales*, which were vastly abundant in site PB, perform different roles in nitrogen cycling, including nitrogen fixation and nitrate reduction [85,86].

## 5. Conclusions

The present study first unveils comprehensive data on the effect of environmental factors, including soil and surface water characteristics, on the soil bacterial community composition of six mangrove forests spanning approximately 660 km along the Upper Gulf of Thailand. Mangrove soils adjacent to the Upper Gulf of Thailand were variable with respect to pH (acidic to slightly alkaline) and had low amounts of OM. Mangrove soil at Kungkrabaen Bay exhibited the significantly highest amounts of OM, total N, total P, total K, and total Mg, whereas mangrove soil at Laemphakbia Promontory had significantly lowest amounts of OM, total N, total Fe, total Cu, and total Zn. Among all sampling sites, high-throughput sequencing attested that the phylum *Proteobacteria* was mostly distributed among all mangrove forest soils, followed by *Desulfobacterota*, *Bacteroidota*, *Chloroflexi, Crenarchaeota*, *Acidobacteriota*, *Firmicutes*, *Myxococcota*, *Gemmatimonadota*, and *Halobacterota*. Even though the bacterial community composition retained its unique pattern based on the number of observed species as well as the bacterial diversity and richness, there were differences in variation and abundance of bacterial taxa among sites. The PcoA plot alienated the bacterial community of soil at Mae Klong Estuary from that of other sites. Soil and surface water parameters significantly affected the bacterial diversity and richness, in either a positive or negative manner, depending on individual classes. Overall, soil total K, total N, and OM were mostly directly connected to the bacterial diversity. NaCl was the only factor that was closely related in the same direction to the number of observed species and bacterial richness and affected the distribution and relative abundance of dominant bacterial classes. The groups of ammonia-oxidizing, sulfate-reducing, and methanogenic bacteria were significant biomarkers that contributed to the soils of mangrove forest environments. The results not only provide informative data regarding the diversification of bacterial community structure in mangrove forests adjacent to the Upper Gulf of Thailand in response to environmental factors but also monitor the heavy metal pollution status of the studied areas. Both aspects are vital to the sustainable development and management of tropical mangrove ecosystems.

## Figures and Tables

**Table 1:** Mangrove forest sites, geographical locations, and sampling dates.

Site Code	Place	District, Province	Sampling Date	Latitude	Longitude
TR	Black Sand Beach	Laemngop, Trat	21 August 2021	12.1723 N	102.4076 E
KA	Kungkrabaen Bay	Thamai, Chanthaburi	21 August 2021	12.5719 N	101.9011 E
JA	Chaopraya Estuary	Phrasamutchedi, Samutprakan	7 August 2021	13.3702 N	99.9919 E
MK	Mae Klong Estuary	Mueang, Samutsongkhram	7 August 2021	13.5997 N	100.5866 E
PB	Laemphakbia Promontory	Laemphakbia, Phetchaburi	14 August 2021	13.0413 N	100.0902 E
PAR	Pranburi forest park	Pranburi, Prachuapkhirikhan	14 August 2021	12.4236 N	99.9813 E

**Table 2:** Mangrove soil physicochemical properties of each sampling site.

Parameter *	Soil Samples from Site					
	TR	KA	JA	MK	PB	PAR
pH	6.28 ± 0.01 c **	5.52 ± 0.02 b	6.91 ± 0.01 d	7.12 ± 0.00 e	7.40 ± 0.01 f	4.98 ± 0.00 a
EC (dS/m)	19.20 ± 0.00 b	39.71 ± 0.01 e	12.33 ± 0.01 a	27.21 ± 0.01 c	31.60 ± 0.00 d	44.51 ± 0.01 f
OM (g/kg)	0.57 ± 0.01 e	1.85 ± 0.19 f	0.35 ± 0.00 c	0.32 ± 0.01 b	0.19 ± 0.00 a	0.52 ± 0.00 d
N (mg/kg)	20.00 ± 0.00 e	45.00 ± 0.01 f	16.00 ± 0.00 d	14.00 ± 0.00 c	9.000 ± 0.00 a	12.60 ± 0.01 b
P (mg/kg)	20.98 ± 0.05 a	90.98 ± 0.14 f	74.43 ± 0.39 e	32.82 ± 0.17 b	72.62 ± 0.21 d	34.77 ± 0.27 c
K (mg/kg)	793.44 ± 5.84 d	1022.06 ± 16.17 e	729.60 ± 14.61 c	732.96 ± 7.90 c	641.78 ± 19.18 b	523.70 ± 11.00 a
Ca (mg/kg)	1686.20 ± 96.88 b	2880.05 ± 12.73 c	1838.63 ± 16.32 b	6715.81 ± 37.57 e	6020.12 ± 125.42 d	972.03 ± 0.51 a
Mg (mg/kg)	1550.79 ± 7.52 b	3614.57 ± 29.35 d	1737.39 ± 116.96 c	1438.67 ± 24.34 ab	1297.53 ± 35.96 a	1440.86 ± 32.79 ab
Cd (mg/kg)	ND ***	ND	ND	ND	ND	ND
Cr (mg/kg)	ND	ND	ND	ND	ND	ND
Ag (mg/kg)	ND	ND	ND	ND	ND	ND
Se (mg/kg)	ND	ND	ND	ND	ND	ND
Cu (mg/kg)	4.40 ± 0.07 c	9.05 ± 0.33 d	11.53 ± 0.17 e	2.97 ± 0.08 b	1.00 ± 0.01 a	3.95 ± 0.06 c
Al (mg/kg)	ND	ND	ND	ND	ND	ND
Ba (mg/kg)	ND	ND	ND	ND	ND	ND
Hg (mg/kg)	ND	ND	ND	ND	ND	ND
Mn (mg/kg)	101.96 ± 2.60 c	5.54 ± 0.17 a	191.14 ± 2.21 e	157.81 ± 6.31 d	105.03 ± 3.65 c	21.75 ± 0.55 b
As (mg/kg)	ND	ND	ND	ND	ND	ND
Zn (mg/kg)	1.92 ± 0.13 b	5.94 ± 0.33 d	21.43 ± 0.31 e	3.76 ± 0.19 c	0.92 ± 0.02 a	5.68 ± 0.09 d
Pb (mg/kg)	ND	ND	ND	ND	ND	ND
Fe (mg/kg)	251.92 ± 9.32 c	273.26 ± 5.37 d	256.25 ± 7.57 cd	119.91 ± 1.57 b	58.01 ± 0.05 a	383.34 ± 11.43 e
Sand (%)	28.92	10.14	24.18	32.04	31.74	58.09
Silt (%)	59.74	56.89	60.39	46.58	58.18	31.24
Clay (%)	11.33	32.97	15.44	21.38	10.08	10.67
Soil texture	Silt Loam	Silty Clay Loam	Silt Loam	Loam	Silt Loam	Sandy Loam

* All values are presented as means ± SD from triplicate samples. ** Values with the same letters in the row are not significantly different (*p* > 0.05) according to Tukey’s test. *** ND, not detected. The site codes are in accordance with those listed in Table 1.

**Table 3:** Mangrove surface water physicochemical properties of each sampling site.

Parameter *	Surface Sample from Site					
	TR	KA	JA	MK	PB	PAR
pH	7.660 ± 0.017 c **	7.536 ± 0.005 a	7.880 ± 0.020 e	8.010 ± 0.010 f	7.603 ± 0.015 b	7.800 ± 0.017 d
NaCl (%)	0.000	1.000 ± 0.000 a	0.000	0.000	3.000 ± 0.000 c	2.000 ± 0.000 b
Cd (mg/L)	ND ***	ND	ND	ND	ND	ND
Cr (mg/L)	ND	<LOQ ***	ND	<LOQ	ND	ND
Ag (mg/L)	ND	0.026 ± 0.001 b	ND	ND	0.011 ± 0.000 a	ND
Se (mg/L)	ND	ND	ND	ND	ND	ND
Cu (mg/L)	0.171 ± 0.006	ND	ND	ND	ND	ND
Al (mg/L)	0.013 ± 0.000 a	0.028 ± 0.001 c	<LOQ	<LOQ	0.019 ± 0.000 b	0.013 ± 0.000 a
Ba (mg/L)	ND	0.014 ± 0.000 a	0.027 ± 0.000 c	0.039 ± 0.001 d	0.019 ± 0.000 b	0.056 ± 0.001 e
Hg (mg/L)	ND	ND	ND	ND	ND	ND
Mn (mg/L)	0.122 ± 0.007 c	0.032 ± 0.001 b	1.296 ± 0.02 d	0.010 ± 0.000 a	<LOQ	<LOQ
As (mg/L)	<LOQ	<LOQ	<LOQ	0.023 ± 0.000	<LOQ	<LOQ
Zn (mg/L)	0.011 ± 0.000 a	<LOQ	<LOQ	0.015 ± 0.000 b	<LOQ	<LOQ
Pb (mg/L)	<LOQ	<LOQ	0.012 ± 0.001 a	<LOQ	0.016 ± 0.000 b	0.018 ± 0.001 c
Fe (mg/L)	ND	ND	ND	ND	ND	<LOQ

* All values are presented as means ± SD from triplicate samples. ** Values with the same letters in the row are not significantly different (*p* > 0.05) according to Tukey’s test. *** ND, not detected; <LOQ, Below the lowest limit of quantitation (LOQ of each element was 0.01 mg/L). The site codes are in accordance with those listed in Table 1.

**Table 4:** Indices of bacterial diversity and richness of mangrove soil from each sampling site.

Soil Samples Form Site	Number of Observed Species *	Diversity Indices *		Richness Indices *	
		Shanon–Weaver	Simpson	Chao1	ACE
TR	7563.66 ± 657.49 a **	11.20 ± 0.25 a	0.99 ± 0.00 a	10,203.01 ± 907.83 a	10,573.65 ± 928.85 a
KA	7496.66 ± 110.56 a	11.27 ± 0.05 a	0.99 ± 0.00 a	9619.66 ± 951.11 a	10,072.83 ± 784.60 a
JA	5881.33 ± 126.47 a	10.63 ± 0.12 a	0.99 ± 0.00 a	7541.69 ± 871.15 a	7872.85 ± 728.95 a
MK	5814.00 ± 2415.35 a	10.04 ± 1.75 a	0.98 ± 0.02 a	7697.23 ± 3638.31 a	8051.35 ± 3787.22 a
PB	6260.00 ± 811.15 a	10.67 ± 0.39 a	0.99 ± 0.01 a	8118.93 ± 1757.09 a	8521.46 ± 1736.08 a
PAR	6295.66 ± 931.50 a	11.05 ± 0.19 a	0.99 ± 0.00 a	7748.19 ± 2109.44 a	7892.24 ± 2140.54 a

* All values are presented as means ± SD from triplicate samples. ** Values with the same letters in the column are not significantly different (*p* > 0.05) according to Tukey’s test. The site codes are in accordance with those listed in Table 1.

**Table 5:** Spearman’s (*r_s_*) correlations between abiotic and biotic factors of each sampling site.

Correlations														
Factors	Correlation	Observed Species	Bacterial Richness	Bacterial Community	Gamma-*Proteobacteria*	*Desulfobacteria*	*Bacteroidia*	*Anaerolineae*	Alpha*-Proteobacteria*	*Bathyarchaeia*	*Acidobacteriae*	*Nitrososphaeria*	*Clostridia*	*Thermoplasmata*
Soil pH	*r_s_*	−0.327	−0.146	−0.508 *	0.569 *	−0.708 **	−0.160	0.522 *	0.058	0.177	−0.226	0.329	0.187	−0.406
	Sig.	0.185	0.564	0.031	0.014	0.001	0.526	0.026	0.820	0.483	0.367	0.183	0.458	0.095
Soil EC	*r_s_*	0.102	0.001	0.322	−0.048	0.540 *	0.551 *	−0.415	−0.139	−0.392	−0.062	−0.396	0.141	−0.007
	Sig.	0.686	0.997	0.193	0.851	0.021	0.018	0.087	0.583	0.108	0.807	0.104	0.578	0.977
Soil OM	*r_s_*	0.602 **	0.408	0.649 **	−0.558 *	0.647 **	0.199	−0.534 *	−0.096	−0.406	0.049	−0.207	−0.414	0.342
	Sig.	0.008	0.093	0.004	0.016	0.004	0.428	0.023	0.705	0.095	0.848	0.409	0.088	0.165
Soil N	*r_s_*	0.556 *	0.398	0.515 *	−0.598 **	0.387	0.141	−0.403	−0.243	−0.347	−0.025	−0.185	−0.394	0.408
	Sig.	0.017	0.102	0.029	0.009	0.113	0.577	0.097	0.330	0.158	0.921	0.462	0.106	0.093
Soil P	*r_s_*	−0.066	−0.143	−0.064	−0.320	0.539 *	0.156	−0.110	−0.598 **	0.091	−0.527 *	−0.463	−0.279	0.680 **
	Sig.	0.794	0.572	0.801	0.195	0.021	0.536	0.665	0.009	0.720	0.025	0.053	0.262	0.002
Soil K	*r_s_*	0.519 *	0.426	0.381	−0.278	0.156	0.245	−0.315	−0.191	−0.478 *	−0.226	−0.082	−0.358	0.154
	Sig.	0.027	0.078	0.119	0.265	0.537	0.328	0.203	0.448	0.045	0.367	0.748	0.145	0.542
Soil Ca	*r_s_*	−0.146	−0.049	−0.259	0.317	−0.492 *	0.404	0.139	−0.282	−0.325	−0.207	−0.146	0.387	−0.232
	Sig.	0.565	0.848	0.299	0.200	0.038	0.097	0.581	0.257	0.188	0.409	0.565	0.113	0.354
Soil Mg	*r_s_*	0.404	0.247	0.383	−0.666 **	0.465	0.065	−0.321	−0.441	−0.143	−0.086	−0.302	−0.397	0.668 **
	Sig.	0.097	0.324	0.117	0.003	0.052	0.798	0.194	0.067	0.570	0.735	0.223	0.103	0.002
Soil Fe	*r_s_*	0.195	0.005	0.356	−0.496 *	0.697 **	0.129	−0.513 *	−0.092	−0.152	0.127	−0.404	−0.234	0.513 *
	Sig.	0.438	0.984	0.147	0.036	0.001	0.610	0.030	0.717	0.548	0.616	0.097	0.349	0.030
Soil Mn	*r_s_*	−0.511 *	−0.360	−0.659 **	0.261	−0.717 **	−0.463	0.529 *	0.073	0.480 *	0.119	0.232	0.207	−0.030
	Sig.	0.030	0.142	0.003	0.295	0.001	0.053	0.024	0.773	0.044	0.639	0.354	0.409	0.906
Soil Cu	*r_s_*	0.199	0.071	0.168	−0.655 **	0.393	−0.293	−0.270	−0.216	0.115	0.038	−0.245	−0.456	0.730 **
	Sig.	0.428	0.779	0.504	0.003	0.107	0.237	0.279	0.390	0.650	0.880	0.327	0.057	0.001
Soil Zn	*r_s_*	−0.137	−0.269	−0.074	−0.516 *	0.410	−0.107	−0.284	−0.404	0.189	−0.032	−0.574 *	−0.210	0.914 **
	Sig.	0.586	0.281	0.769	0.028	0.091	0.671	0.253	0.096	0.452	0.900	0.013	0.403	0.000
Water pH	*r_s_*	−0.467	−0.366	−0.467	0.114	−0.551 *	−0.233	0.215	0.061	0.302	0.411	−0.118	0.416	0.107
	Sig.	0.051	0.135	0.051	0.653	0.018	0.353	0.392	0.810	0.224	0.090	0.641	0.086	0.671
Water NaCl	*r_s_*	−0.143	−0.157	−0.023	0.263	0.370	0.230	0.017	−0.023	0.003	−0.363	−0.023	0.003	−0.183
	Sig.	0.571	0.535	0.927	0.291	0.131	0.359	0.948	0.927	0.990	0.139	0.927	0.990	0.467
Water Al	*r_s_*	0.360	0.253	0.438	−0.054	0.652 **	0.418	−0.270	−0.168	−0.327	−0.450	−0.095	−0.285	−0.039
	Sig.	0.143	0.310	0.069	0.833	0.003	0.085	0.278	0.504	0.185	0.061	0.709	0.252	0.878
Water Mn	*r_s_*	0.221	0.181	0.090	−0.477 *	−0.020	−0.389	0.038	−0.119	0.196	0.072	0.069	−0.371	0.442
	Sig.	0.378	0.472	0.721	0.045	0.937	0.110	0.881	0.638	0.436	0.778	0.784	0.129	0.066
Water As	*r_s_*	−0.129	−0.043	−0.158	0.244	−0.589 *	0.330	−0.043	−0.101	−0.330	0.273	−0.244	0.560 *	−0.187
	Sig.	0.609	0.865	0.531	0.329	0.010	0.180	0.865	0.691	0.180	0.273	0.329	0.016	0.458
Water Zn	*r_s_*	0.189	0.267	0.108	0.193	−0.634 **	0.122	0.007	0.234	−0.345	0.441	0.197	0.349	−0.475 *
	Sig.	0.452	0.284	0.671	0.443	0.005	0.628	0.977	0.351	0.161	0.067	0.434	0.156	0.046
Water Pb	*r_s_*	−0.561 *	−0.526 *	−0.419	0.221	0.140	−0.288	0.263	0.104	0.537 *	−0.081	0.048	0.072	0.027
	Sig.	0.015	0.025	0.083	0.379	0.580	0.246	0.292	0.681	0.022	0.749	0.851	0.776	0.917

* Correlation is significant at the 0.05 level. ** Correlation is significant at the 0.01 level.

**Table 6:** Percentages of OTUs of top ten most abundant bacterial classes present in each sampling site.

Bacterial Class	Bacterial Number in Soil Sample from Site *					
	TR	KA	JA	MK	PB	PAR
Gamma-*Proteobacteria*	19.34 ± 0.70 a **	16.52 ± 2.53 a	17.68 ± 5.31 a	28.93 ± 14.55 a	29.54 ± 3.46 a	19.66 ± 1.90 a
*Desulfobacteria*	3.16 ± 1.17 a	15.66 ± 1.30 c	3.73 ± 0.65 a	1.87 ± 0.68 a	3.51 ± 0.08 a	8.09 ± 0.34 b
*Bacteroidia*	5.29 ± 2.07 ab	10.78 ± 0.52 b	2.91 ± 1.49 a	8.64 ± 3.35 b	6.10 ± 1.45 ab	6.24 ± 2.22 ab
*Anaerolineae*	7.33 ± 1.44 a	4.24 ± 0.76 a	8.51 ± 2.01 a	6.35 ± 3.16 a	8.99 ± 0.94 a	5.85 ± 1.24 a
Alpha-*Proteobacteria*	8.74 ± 1.41 a	5.79 ± 0.10 a	6.27 ± 0.50 a	6.93 ± 2.18 a	7.53 ± 1.40 a	7.32 ± 1.49 a
*Bathyarchaeia*	1.04 ± 0.37 ab	0.49 ± 0.13 a	9.10 ± 0.24 c	0.72 ± 0.33 ab	1.59 ± 0.71 b	1.14 ± 0.16 ab
*Acidobacteriae*	3.93 ± 2.39 a	0.61 ± 0.12 a	3.17 ± 4.48 a	3.15 ± 0.15 a	0.90 ± 0.78 a	4.20 ± 3.26 a
*Nitrososphaeria*	6.18 ± 0.41 c	0.28 ± 0.04 a	0.56 ± 0.60 a	0.42 ± 0.23 a	2.93 ± 0.31 b	0.44 ± 0.21 a
*Clostridia*	0.91 ± 1.45 ab	0.15 ± 0.03 a	0.43 ± 0.57 a	3.95 ± 2.61 b	0.39 ± 0.19 a	0.74 ± 0.57 ab
*Thermoplasmata*	0.26 ± 0.28 a	2.12 ± 0.65 b	3.39 ± 0.87 c	0.58 ± 0.88 a	0.16 ± 0.06 a	1.29 ± 0.19 ab

* All values are presented as means ± SD from triplicate samples. ** Values with the same letters in the row are not significantly different (*p* > 0.05) according to Tukey’s test. The site codes are in accordance with those listed in Table 1.

## Data Availability

All output data generated or analyzed during this study are provided in this published article. Sequence data can be retrieved from the Sequence Read Archive of the NCBI under BioProject accession number PRJNA856803.

## Supplementary Materials

The following supporting information can be downloaded at: https://www.mdpi.com/article/10.3390/biology11121787/s1, Figure S1: Rank abundance curve of observed species in mangrove forest soils; Figure S2: Canonical correlation analysis (CCA) for evaluating the relationship between environmental factors and bacterial diversity and richness. Redundancy analysis plot showing the relationship between physicochemical parameters of soil (s) and surface water (w) and characteristics of bacterial community structures including diversity, richness, and number of observed species (Species) (A); Ternary plots of sampling site grouping in cases of the presence of NaCl (B) and the absence of NaCl (C). Color and size of each circle denote the dominant class and its relative abundance, respectively; Table S1: Raw sequence and taxonomically classified sequence data of triplicate samples.
