# Supplementary material for: Insights into Bacterial Communities and Diversity of Mangrove Forest Soils along the Upper Gulf of Thailand in Response to Environmental Factors
Source: Biology (Basel). 2022 Dec 8;11(12):1787. doi: 10.3390/biology11121787 (PMC9775068; doi:10.3390/biology11121787)
Supplement: Supplementary file 1 [file biology-11-01787-s001.zip › Supplemantary Table.pdf]

Supplementary Table S1. Raw sequence and taxonomically classified sequence data of triplicate samples.

Sample name	Total raw reads	Combined reads	Percent efficiency (%)	Total tag	Taxon Tag	Unclassified Tag	Unique Tag	Observed species
MK1	136,008	133,168	97.91	116887	103457	8601	4829	3656
MK2	125,666	122,954	97.84	91177	80248	90	10839	8337
MK3	137,034	134,189	97.92	102840	91590	176	11074	8366
KA1	132,400	128,063	96.72	72462	64399	755	7308	6711
KA2	139,470	135,637	97.25	90960	78759	756	11445	7091
KA3	116,525	113,184	97.13	91241	80128	1129	9984	7030
TR1	125,116	121,123	96.81	90768	80286	71	10411	8019
TR2	125,931	123,581	98.13	97508	86116	127	11265	7957
TR3	131,265	126,894	96.67	73097	64871	151	8075	6240
JA1	97,031	93,804	96.67	53210	46302	252	6656	6175
JA2	125,366	123,107	98.2	83650	74753	295	8602	7141
JA3	126,294	124,010	98.19	95507	82345	301	12861	8529
PAR1	71,751	70,278	97.95	87735	72297	89	15349	8788
PAR2	110,465	107,217	97.06	96869	80943	130	15796	8975
PAR3	130,455	127,974	98.1	77509	64223	187	13099	8553
PB1	123,079	119,740	97.29	91325	78696	385	12244	9596
PB2	129,158	126,117	97.65	94299	82412	290	11597	7945
PB3	98,856	95,436	96.54	91923	78800	297	12826	9372
