# Supplementary material for: Insights into Bacterial Communities and Diversity of Mangrove Forest Soils along the Upper Gulf of Thailand in Response to Environmental Factors
Source: Biology (Basel). 2022 Dec 8;11(12):1787. doi: 10.3390/biology11121787 (PMC9775068; doi:10.3390/biology11121787)
Supplement: Supplementary file 1 [file biology-11-01787-s001.zip › Supplementary Figure.pdf]

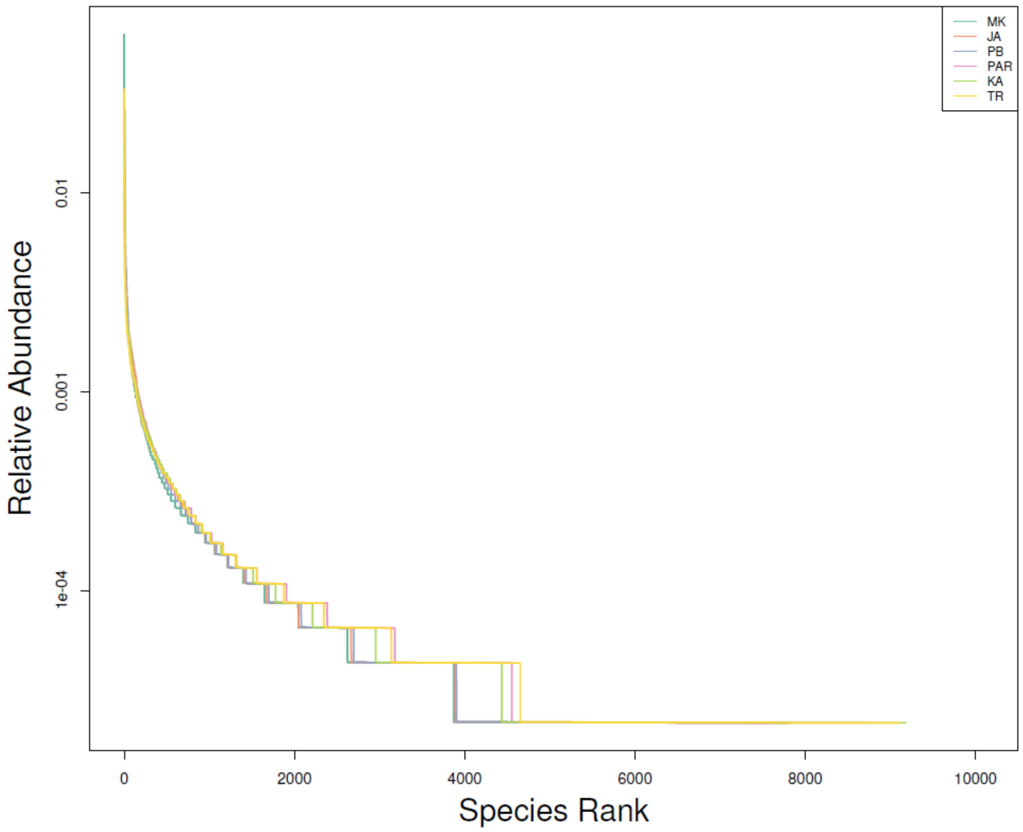

Supplementary Figure S1. Rank abundance curve of observed species in mangrove forest soils. The site codes are in accordance with those listed in Table 1.

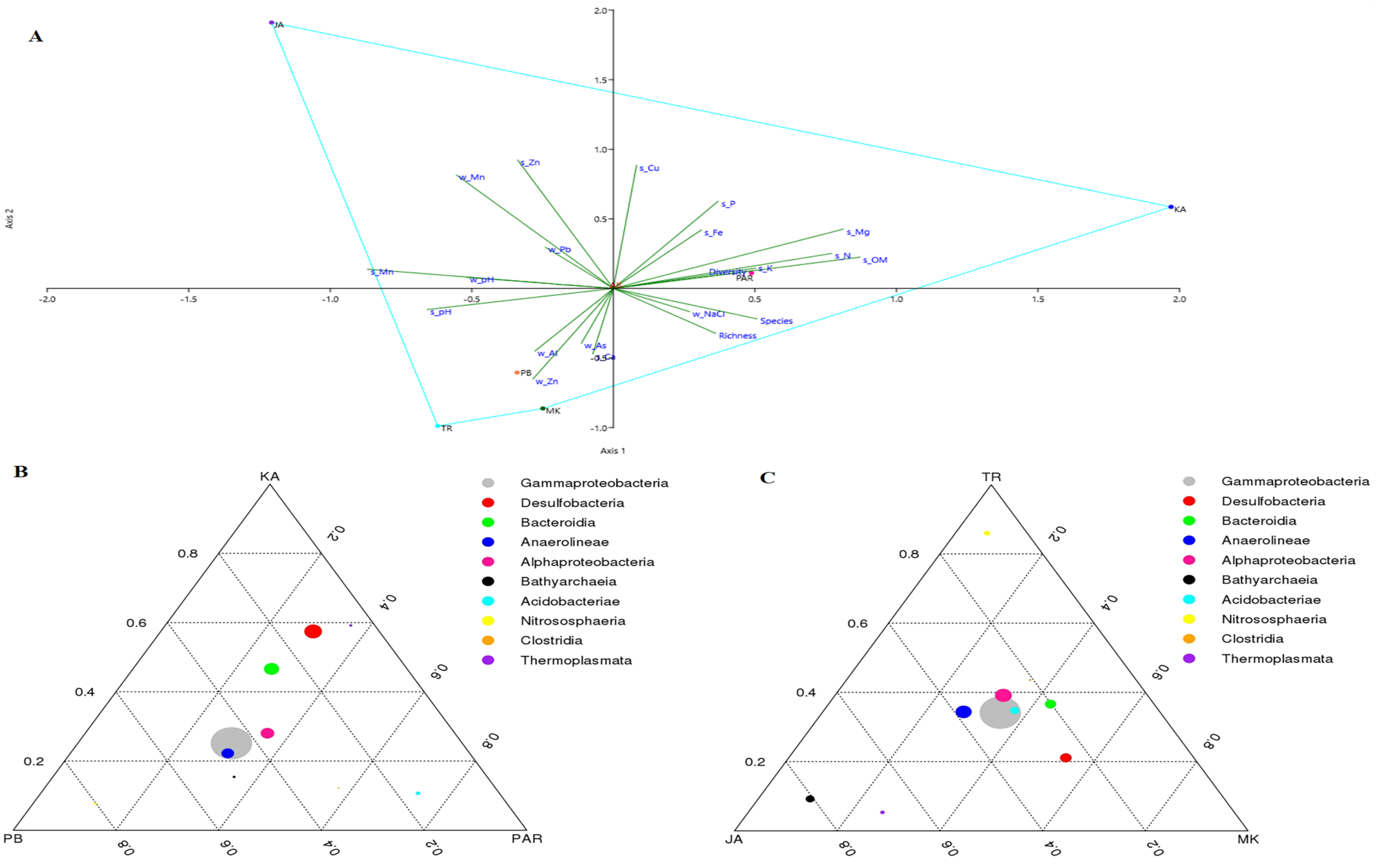

Supplementary Figure S2. Canonical correlation analysis (CCA) for evaluating the relationship between environmental factors and bacterial diversity and richness. Redundancy analysis plot showing the relationship between physicochemical parameters of soil (s) and surface water (w) and characteristics of bacterial community structures including diversity, richness, and number of observed species (Species) (S2A); Ternary plots of sampling site grouping in cases of the presence of NaCl (S2B) and the absence of NaCl (S2C). Color and size of each circle denote the dominant class and its relative abundance, respectively.
